# Retraction: Screening for Postpartum Post-traumatic Stress Disorder (PTSD) Using the Kurdish Version of the PTSD Checklist for DSM-5 (PCL-5)

**DOI:** 10.7759/cureus.r209

**Published:** 2025-12-05

**Authors:** Renaz O Ibrahim, Shahla Alalaf, Noor Elebiary

**Affiliations:** 1 Obstetrics and Gynecology, Maternity Teaching Hospital, Ministry of Health, Erbil, IRQ; 2 Obstetrics and Gynecology, College of Medicine, Hawler Medical University, Erbil, IRQ; 3 Obstetrics and Gynecology, Ninewells Hospital, Dundee, GBR

This article has been retracted by the Editors-in-Chief due to major methodological and data integrity problems that undermine the reliability of its findings. The reported recruitment numbers cannot be verified, and Table 3 contains impossible obstetric classifications, including treating induction of labor as a separate delivery mode and presenting an implausibly low cesarean rate. The relationship between postpartum hemorrhage and blood transfusion also contradicts expected clinical patterns. The authors' explanations did not resolve these issues, and the study does not meet the standards required for scientific validity. The authors disagree with the decision to retract.

